# E-Cigarettes as a Growing Threat for Children and Adolescents: Position Statement From the European Academy of Paediatrics

**DOI:** 10.3389/fped.2021.698613

**Published:** 2021-10-04

**Authors:** Andrew Bush, Agnieszka Lintowska, Artur Mazur, Adamos Hadjipanayis, Zacchi Grossman, Stefano del Torso, Pierre-André Michaud, Svitlana Doan, Ivanna Romankevych, Monique Slaats, Algirdas Utkus, Łukasz Dembiński, Marija Slobodanac, Arunas Valiulis

**Affiliations:** ^1^Imperial College Centre for Paediatrics and Child Health, National Heart and Lung Institute, Royal Brompton Harefield NHS Foundation Trust, London, United Kingdom; ^2^Department of Health Promotion, Faculty of Health Science, Wroclaw Medical University, Wroclaw, Poland; ^3^Institute of Medical Sciences, Medical College, University of Rzeszów, Rzeszow, Poland; ^4^European Academy of Paediatric (EAP/UEMS-SP), Brussels, Belgium; ^5^Medical School, European University of Cyprus, Nicosia, Cyprus; ^6^Adelson School of Medicine, Ariel University, Ariel, Israel; ^7^Maccabi Health Services, Tel Aviv, Israel; ^8^Pediatra di Famiglia ULSS 16, Padua, Italy; ^9^Faculté de Biologie et de Médecine, Université de Lausanne, Lausanne, Switzerland; ^10^Department of Public Health and Microbiology, Kyiv Medical University, Kyiv, Ukraine; ^11^Shupyk National Medical Academy of Postgraduate Education, Kyiv, Ukraine; ^12^Pediatric Pulmonology, Sophia Children's Hospital, Rotterdam, Netherlands; ^13^Department of Human and Medical Genetics, Institute of Biomedical Sciences, Vilnius University Medical Faculty, Vilnius, Lithuania; ^14^Department of Pediatric Gastroenterology and Nutrition, Medical University of Warsaw, Warsaw, Poland; ^15^Department of Pediatrics, Health Centre Ðakovo, Ðakovo, Croatia; ^16^Clinic of Children's Diseases, Institute of Clinical Medicine, and Department of Public Health, Institute of Health Sciences, Vilnius University Medical Faculty, Vilnius, Lithuania

**Keywords:** e-cigarettes, electronic nicotine delivery devices, heated tobacco products, children, adolescents, statement, European academy of paediatrics

## Abstract

As the tobacco epidemic has waned, it has been followed by the advent of electronic nicotine delivery devices (ENDS) primarily manufactured by the tobacco industry to try to recruit replacements for deceased tobacco addicts. This document sets out the ten recommendations of the European Academy of Paediatrics (EAP) with regard to e-cigarettes and children and young people (CYP). The EAP notes that nicotine is itself a drug of addiction, with toxicity to the foetus, child and adult, and were ENDS only to contain nicotine, their use to create a new generation of addicts would be rigorously opposed. However, e-cigarettes include numerous unregulated chemicals, including known carcinogens, whose acute and long term toxicities are unknown. The EAP asserts that there is incontrovertible evidence that the acute toxicity of e-cigarettes is greater than that of “traditional” tobacco smoking, and a variety of acute pulmonary toxicities, including acute lung injuries, have been recorded due to e-cigarettes usage. The chronic toxicity of e-cigarettes is unknown, but given the greater acute toxicity compared to tobacco, the EAP cannot assume that e-cigarettes are safer in the long term. The high uptake of e-cigarettes by CYP, including under-age children, is partly fuelled by deceitful marketing and internet exposure, which is also unregulated. Although proposed as aids to smoking cessation, there is no evidence that e-cigarettes add anything to standard smoking cessation strategies. In summary, the EAP regards these devices and liquids as very dangerous, and ineluctably opposed to their use, and their direct or indirect marketing.

## Introduction

The adverse health effects of tobacco, which extend right across the developmental course from transgenerational to antenatal through childhood to old age are well-documented. As legislation increasingly restricts the sale of tobacco, and of course as the current generation of people addicted to tobacco dies off prematurely (1, 2), the industry it seems has to find ways to recruit new addicts in order to make money. A new initiative has come in the form of a variety of heated products for inhalation, some but not all of which contain nicotine. These are advertised as being a safer and more socially acceptable form of smoking. This statement sets out the reasons why the European Academy of Paediatrics (EAP) believes these new developments are potentially more harmful to children and young people (CYP) even than “traditional” tobacco smoking. This particularly applies to nicotine containing products, but also to nicotine free liquids. The consequence of the evidence that will be marshalled here is that the EAP will make ten recommendations on the approach to these products, which build on those published earlier by EAP and other international Societies.

The context of these recommendations is the truly terrifyingly rapid embracing of these products by young people; the hugely increasing use of social media and manipulations thereof by the tobacco industry in particular, who are the major suppliers of these products; their acquisition by under-age children; and the likelihood that their use will lead to a new generation of nicotine addicts. EAP cannot tolerate allowing any of these consequences unchallenged.

## What are E-Cigarettes?

These come in many shapes and sizes, including disguised as pipes, cigars, and incredibly, even metered dose inhalers. Essentially, they consist of a battery, a reservoir which is pre-filled or refillable which holds the liquid, which usually contains nicotine. There is a heating element or an atomizer, and a mouthpiece through which the user puffs. The device heats a liquid into an aerosol that is inhaled by the user. E-liquids usually contain propylene glycol or glycerin as a solvent for nicotine and a multiplicity of flavouring and other chemicals.

The first point to be made is that there are thousands of vaping liquids which can be obtained commercially, each containing multiple chemicals in different combinations. Before an inhaled medicinal product is brought on the market, extensive safety testing is mandated for the best and most obvious of reasons. Secondly, the lungs are vulnerable by the inhalational route; recent examples are the devastating outbreak of interstitial lung disease in Korea caused by humidifier disinfectant (3), and the common occurrence of hypersensitivity pneumonitis in India from fungal allergens in air coolers (4). Finally, the point must be stressed that just because it is safe to eat a substance, does not mean it is safe to inhale it; bakers who develop asthma due to flour inhalation can eat wheat containing products with impunity.

It should also be noted that the vast majority of these devices are manufactured by the tobacco industry, whose track record of concealing and obfuscating data about tobacco safety is truly horrendous. It would be unwise to trust any reassurance from such a source. “Fool me once, shame on you; fool me twice, shame on me” as the old Italian proverb states.

## Nicotine as a Substance of Addiction

Nicotine is a pyridine alkaloid found in tobacco and it is contained in cigarettes, other tobacco products and electronic nicotine delivery systems (ENDS). It has been unambiguously proven to possess great psychoactive properties leading to addiction (5). Nicotine is well-absorbed through the respiratory and gastrointestinal tracts, and the skin. Its highest concentration is found in the brain, kidneys, gastric mucosa and adrenal gland (5, 6). After being inhaled with smoke or in an e-cigarette aerosol nicotine enters the lungs and is rapidly (15–30 s) absorbed (7, 8) in the pulmonary venous circulation and thence to the systemic arterial circulation and thus the central nervous system (CNS) (5). Nicotine binds to nicotinic acetylcholine receptors (nACHRs) which are unevenly distributed in the human body. The receptors are especially found in the brain, neuromuscular junctions, autonomic ganglia, and adrenal medulla (9). Within the brain, nACHRs receptors are especially located in the hippocampus, hypothalamus, reticular formation and cerebral cortex, and there is predominant signalling through the mesolimbic or “reward pathway” in the CNS. When nicotine binds to the nACHR receptors, there is stimulation of dopamine neurons in the ventral tegmental area (VTA), which activate the “reward pathway” [the nucleus accumbens, amygdala, and the dorsolateral prefrontal cortex and orbitofrontal cortex (5–7)]. This leads to the consumer experiencing a momentary burst of energy or relaxation (a decrease of stress and anxiety) depending on their current physical and mental state. This also results in rapid release of glucose in the blood, a slight increase of systemic arterial blood pressure and an increased respiratory rate. The psychotropic effects lead to an eagerness to repeat this exposure as it is pleasurable, especially to adolescents. However, repeated exposures lead to nicotine adaptation and gradual development of physical and mental addiction. This reduces the initially positive experiences of taking nicotine and leads to urges to continue using nicotine products (10). Consumption of several cigarettes a day will lead to continuous exposure to nicotine, for 24 h per day (7, 11, 12). Smoking one cigarette causes a nicotine concentration in the blood of 5–30 ng ml. The blood level increases during the act of smoking, reaching the highest point at the end of the cigarette. After smoking, the level of nicotine in the blood drops sharply for about 20 min, with a half-life of 8 min, but it is detectable in the body for another 2 h. Smoking another cigarette causes the same reaction, nicotine increasing to a peak at the end of smoking and then dropping sharply (5, 7, 11). ENDS supply nicotine by vaping in a similar way to conventional cigarettes, through inhalation. Nicotine concentrations are similar in smokers and vapers (13, 14). Brain nicotine levels are lower in vapers than in cigarettes smokers (15), but the urine and salivary nicotine concentrations are similar in both groups (16, 17). The effects are device specific—some ENDS, for example JUULs, are carefully engineered to ensure a nicotine surge, likely increasing the risk of addiction. Furthermore, frequently the actual nicotine concentrations exceed those on the manufacturer's label. Studies in animal models have shown that the potential for adverse effects of using ENDS nicotine health products is large, despite widespread belief that they are less harmful than traditional cigarettes. There are also reports indicating a greater addictive potential of ENDS than traditional cigarettes (2, 18), which contradicts the industry concept that they are an aid to quitting conventional cigarettes.

About 80% of adult smokers report that they started smoking before they were 18 years old (19–22). Studies show that it is most common to start smoking between 11 and 13 years of age, leading to addiction before reaching adulthood (23–25). Initially it was considered that in order to get addicted it was necessary to smoke an average of 20 cigarettes a day for a long period of time (26). However, numerous scientific reports indicate that especially adolescents can become nicotine/cigarette dependent in a shorter period of time, much more rapidly than adults, and despite using nicotine products irregularly and in lesser quantities (22, 26, 27). An increased susceptibility to addiction in adolescence is also shown by animal studies (28–30). Addiction may occur within 4 weeks of smoking the first cigarette or even earlier (23–25, 31). Some studies indicate that a teenager can get addicted to nicotine even after a single exposure to a nicotine product (27, 32).

Withdrawal symptoms become increasingly significant with greater use of these nicotine-containing products. The user has increasing urges to smoke or vape, which are not easy to ignore. With longer and continuous usage, the urge becomes harder and then impossible to ignore. The user cannot concentrate on anything but using a nicotine product in order to function normally (20, 26, 33–36).

In summary, even were vaping to result solely in nicotine exposure, it would be extremely harmful to children and young people (CYP), and should therefore be vigorously opposed.

## Nicotine as a Harmful Substance in its Own Right

The adverse effects of nicotine are well-documented (17, 37) and will only briefly be summarised here. EAP accepts that the effects of human smoking, as opposed to controlled experiments of nicotine exposure in animals, may not be due to nicotine but to some other substance contained in tobacco, and thus not relevant to e-cigarettes; however the toxicology studies need to be carefully considered before e-cigarettes are acquitted. The adverse effects of smoking by pregnant women are well-known, especially the association with premature birth and small for gestational age babies. Animal experiments have clearly implicated nicotine in causing changes in foetal lung structure and cord blood immunological function and reducing birth weight, as well as sensitising the foetus to later adult-life adverse exposures. Lung structural abnormalities include increased collagen deposition in the developing lung; increased MUC5AC and 5B expression; loss of the alveolar tethering points and hence airway instability; and dysanaptic airway growth, with abnormally long airways leading to airway obstruction and bronchial hyper-responsiveness in the newborn rat, independent of the presence of infection or allergic inflammation. A number of points should be made relevant to human health; early impairment of lung function is associated with increased asthma risk up to and including the fourth decade of life, with reduction in airway calibre and wall thinning in the third decade; in three studies, airway hyper-responsiveness shortly after birth was strongly associated with adverse respiratory outcomes in the first two decades of life; and dysanaptic airway growth is associated with worse asthma outcomes, particularly in the obese. Early airflow obstruction tracks into at least the sixth decade and is a risk factor for chronic obstructive pulmonary disease. Finally, airflow obstruction is associated with premature all cause morbidity and mortality, as well as adverse respiratory outcomes (38). Other structural effects of maternal nicotine exposure in animal studies include failure of secondary septation and premature emphysema. Human studies also suggest that smoking is associated with increased thickness of airway smooth muscle. Immunological consequences of smoking in pregnancy as studied in cord blood include increased mononuclear cell reactivity to allergens; reduced interleukin IL-10 and Toll-like receptor function; and reduced IL13 production, this last may be associated with subsequent early viral induced wheeze (1, 39). Finally, maternal smoking sensitises the foetus to adverse effects if the young person subsequently smokes; and early disadvantage, especially including passive smoke exposure, is associated with more rapid adult lung function decline, and a greater susceptibility to adult life occupational exposures (40, 41).

There are also important transgenerational effects of smoking. Two large studies have demonstrated that if a grandmother smokes, irrespective of whether her daughter, smokes, her grandchildren are more likely to develop asthma. Also, there is a strong correlation between low parental lung function (which is likely related at least in part to smoking) and offspring low spirometry [which is associated with bad outcomes (38)].

Chronic nicotine use leads to cardiovascular and neurodegenerative disease, and cancer (22, 42–45), in addition to the adverse effects on the foetus. The mechanism of nicotine damage to blood vessels is endothelial injury and initiation of thrombotic, inflammatory, and oxidative stress processes (35). The association of the use of tobacco products by young people with an abnormal lipid profile in adulthood (initiated in adolescence) as well as the occurrence of coronary atherosclerosis is well-known. Tobacco use in adolescence has also been shown to be associated with occurrence of abdominal aortic aneurysm in early adulthood (17, 46). Nicotine exposure or intake can also lead to impaired brain development in children and adolescents, causing learning difficulties, as well as increased risk of anxiety disorders (25, 47–49).

Smoking cigarettes by CYP and the use of other nicotine products may also increase the risk of other addictions, including marijuana and other drugs. Many studies indicate that using products with nicotine may be “paving the way” for other psychoactive substances such as alcohol or drugs in the future (25, 50–53). Studies of Polish and Ukrainian youth have shown a high correlation between the use of cigarettes and other substances of abuse (*r* = 0.6) (54). The relationship is bi-directional, with nicotine addiction encouraging substance abuse and cannabis leading to nicotine addiction (55). However, we should not be side-tracked into sterile arguments about whether e-cigarettes are a gateway to smoking or anything else; they are sufficiently harmful in their own right that every effort must be made to keep them out of the hands of children and young people.

## Other Exposures from E-Cigarettes and Their Consequences

The data leads inexorably to two conclusions. The first is that e-liquids are unregulated, and contain many different chemicals for which toxicity is unknown. These include known carcinogens, and bacterial and fungal products. The known end-organ effects on the lung include the generation of oxidative stress and impairment of innate immune and anti-viral defences, reviewed in detail elsewhere (49). It would be naïve to think that there are not more adverse effects to be discovered, given the multiplicity of chemicals and chemical combinations that are being inhaled. Finally, it should be noted that the adverse effects of passive tobacco exposure are well-known, and there is evidence that passive exposure to e-cigarettes results in the bystander absorbing toxic compounds; the health effects of passive vaping have not been explored in detail, but it is difficult to believe they will be anything other than adverse.

## Adverse Consequences of E-Cigarette Use

### Acute Toxicity of E-Cigarettes

E-cigarette and vaping induced lung injury (EVALI) has become an epidemic. Elsewhere it has been argued that the EVALD (E-cigarette and vaping induced lung *disease*) is a better term, because although acute lung injury is certainly one result of e-cigarette use, there are many others, including lipoid pneumonia, organising pneumonia, eosinophilic pneumonia acute pulmonary haemorrhage and nodular lung disease, some of which are fatal (1, 16, 17, 22). The definition of EVALI/EVALD is still debated. Current definitions exclude cases with a pre-existing lung disease or isolate of a known respiratory pathogen, but this may not be logical, because it is at least feasible that the effects of these insults might be worsened by e-cigarettes. Definitions also mandate a history of vaping, but there is at least a possibility that passive exposure may cause acute and also chronic toxicity.

It is clearly important not to overcall a diagnosis of EVALI/EVALD, and there is not always good agreement between pathologists and clinicians. This is in part because diffuse alveolar damage is a non-specific response to lung injury, and also because, as a result of admission to Intensive Care, there may be superadded iatrogenic changes such as ventilator associated pneumonia and barotrauma. Notwithstanding, it is clear that many hundreds of unequivocal cases of EVALI/EVALD have been reported, many of which have been fatal or caused long term lung damage.

Finally, an important practise point is that paediatricians should always consider the possibility of e-cigarette usage as the cause of an unusual respiratory disease.

### Chronic Toxicity of E-Cigarettes

There is no question but that the acute toxicity of e-cigarettes far exceeds that of tobacco; that fact of itself means that bland assertions that e-cigarettes are “95% safer than tobacco” are ridiculous. It took decades before it was appreciated that cigarette smoking caused lung cancer, and many years before the incontrovertible evidence was widely accepted. Even today, we are making new discoveries about the long-term hazards of smoking. We simply cannot be complacent about the long term consequences of the inhalation of e-cigarettes and heated tobacco products, and, given the greater acute effects of vaping, we must assume until proven otherwise, that the long term effects are worse as well. It is not the role of the Academy to prove these devices are unsafe; it is up to the industry to prove they are safe, *if they can*.

## Smoking Cessation

Since it is well-known that many under age children are already using and have become addicted to cigarettes, so effective smoking cessation strategies are an important concern of the Academy. In summary, there is no evidence of superiority of e-cigarettes over standard techniques such as nicotine replacement therapy and pharmacological methods such as buprion and varinecycline. Many of the apparently impressive data on the use of e-cigarettes as an aid to smoking cessation on closer inspection show that those who have “quit” smoking have continued to use e-cigarettes, thus merely exchanging one dangerous addiction for another. If the Industry was really serious in wanting to help smokers quit, they would have produced a graded series of liquids with reducing concentrations of nicotine, so the addict could gradually be weaned off this chemical, but rather their strategy is to increase exposure by generating nicotine surges.

A recent Cochrane review (56) stated that there was moderate certainty that quitting was more likely to be successful using nicotine containing e-cigarettes as compared to standard nicotine replacement therapy or e-cigarettes which did not contain nicotine. They recorded no evidence harm of e-cigarettes in a 2 year follow-up period (“there is none so blind as those who will not see”) and included no comparisons with pharmacological therapy as an aid to smoking cessation. The review was heavily criticised because of links between the industry and an author and one of the reviewers, and also there is no support for the conclusions in population studies (17). Furthermore, the point was made that many continue to use on e-cigarette after “quitting” and harm data was not properly considered. There is no reason to change practise on the basis of this review.

The Academy recognises that there may be a very small number of smokers who are unable to quit by any other means, for whom e-cigarettes may offer the only hope of breaking their addiction, but for the vast majority of smokers, quitting is best achieved without using e-cigarettes.

## How are E-Cigarettes Marketed?

There is a strong sense of déjà vu when looking at advertisements for e-cigarettes. The themes are so similar, including those focused on freedom, rebellion, and glamour. This of itself gives the lie to the idea that these are marketed for smoking cessation; who has seen a nicotine patch advertised attached to a shapely naked arm? Electronic cigarette products have also been marketed with a number of unsubstantiated health and cessation messages, both on radio and television. The use of social media (e.g., YouTube, Instagram, and Facebook) is particularly concerning, not just for advertising but also as a source of these products, which can readily be purchased through websites. One study analysed 245,894 posts over a 4 years period (57). Pro-vaping hashtags were used thousands of times more frequently than FDA warnings; indeed, after such warnings were issued, there were more not fewer “likes” about vapes. Frequent themes were pods (which give a nicotine surge, above), flavours, devices and user experience; the real cost of vaping hardly rated a mention. Worryingly, under-age followers were recorded. It is not possible to know how many of these posts were planted by the Industry, given the Byzantine obscurity and anonymity tolerated in social media. The use of these resources to promote and supply to CYP is of enormous concern to the Academy.

## Recommendations

The data summarised above inexorably leads the European Academy of Paediatrics to make the following recommendations, which closely align with and amplify those of other international groups (2, 56, 58–60), such as the American Academy of Paediatrics, the European Respiratory Society, the American Thoracic Society, and the International Federation of Respiratory Societies.

1. The European Academy of Paediatrics proposes that e-cigarettes should be considered to be dangerous until proven otherwise. These products comprise literally thousands of liquids containing tens of thousands of chemicals, for almost all of which neither the short or long term toxicity is known. As with medicial products for inhalation, the onus is on the manufacturers to prove the safety of these products, not on physicians to prove that they are unsafe.
2. The European Academy of Paediatrics considers that e-cigarettes are a gateway to nicotine addiction. The Academy will not enter into a debate about whether or not they are a gateway to smoking because this is irrelevant; nicotine addiction and its multisystem health consequences in young people must be prevented, irrespective of whether these products lead on to smoking tobacco.
3. The European Academy of Paediatrics believes that the addition of flavourings to e-liquids is a deliberate attempt by the industry to enhance the use of these products, and cannot in any way be said to aid their utility as aids to smoking cessation. The Academy calls for an immediate ban on the addition of flavourings to e-liquids.
4. E-cigarettes, whether or not they contain nicotine, contain chemicals whose acute and chronic toxicity is either unknown, or known to be harmful, including being carcinogenic, pro-inflammatory and immunosuppressive. The European Academy of Paediatrics insists that children and young people must be protected from the effects of these chemicals, and that includes protection from passive exposure to these products.
5. Devices used for inhaling these products can also be used for inhaling other substances of addiction, including cannabinoids, which add to the toxicity of these products. The European Academy of Paediatrics considers that children and young people should not be given access to such devices.
6. There is overwhelming evidence that the acute toxicity of e-cigarettes is far in excess of that of conventional tobacco products. The European Academy of Paediatrics insists that children and young people must be protected from the multiple acute lung diseases caused by e-cigarettes.
7. The potential medium and long term toxicity of e-cigarettes is as yet unknown because of insufficient time to study them; but given that acute toxicity is greater than tobacco, the recommendation of the European Academy of Paediatrics is that until proven otherwise the long term toxicity of these liquids must be considered a greater threat even than that of tobacco.
8. The European Academy of Paediatrics notes the overwhelming scientific evidence that e-liquids not merely have overlapping toxicity in numerous experimental studies with that of tobacco, but also exerts additional harmful effects. The Academy recommends that e-liquids should not be considered a watered down version of tobacco, but to be toxic in novel ways in their own right.
9. Children and young people should be protected by legislation from exposure to e-cigarettes. The European Academy of Paediatrics recognises the huge benefits of such legislation in curbing tobacco smoking and ameliorating its adverse effects, both on smokers and those who passively inhale, including the foetus. The Academy recommends that e-cigarettes are treated in exactly the same way in terms of legislation as conventional tobacco products, by banning their use in public places and enclosed spaces such as cars, banning all advertising, insisting on plain packaging with health warnings, and the introduction of stringent penalties for the sale of these products to under-age children and young people.
10. The European Academy of Paediatrics notes with profound alarm that social media is being used to entice young people including under-age children to start and continue e-cigarette use, and to obtain access to these products. The Academy recommends that social media companies be compelled to take responsibility for this, and take steps to prevent this happening in the future.

## Data Availability Statement

The original contributions presented in the study are included in the article/supplementary material, further inquiries can be directed to the corresponding authors.

## Author Contributions

AB, AL, AM, AH, ZG, ST, P-AM, AU, and AV: study design. AB, AL, AH, ZG, ST, and AV: data collection. AB, AL, AM, AH, ZG, ST, P-AM, SD, IR, MSla, AU, ŁD, MSlo, and AV: data analysis and interpretation, manuscript preparation, and critical revision. All authors read and approved the final manuscript.

## Conflict of Interest

The authors declare that the research was conducted in the absence of any commercial or financial relationships that could be construed as a potential conflict of interest.

## Publisher's Note

All claims expressed in this article are solely those of the authors and do not necessarily represent those of their affiliated organizations, or those of the publisher, the editors and the reviewers. Any product that may be evaluated in this article, or claim that may be made by its manufacturer, is not guaranteed or endorsed by the publisher.
